# Individual synaptic vesicles from the electroplaque of *Torpedo californica*, a classic cholinergic synapse, also contain transporters for glutamate and ATP

**DOI:** 10.1002/phy2.206

**Published:** 2014-01-28

**Authors:** Huinan Li, Mark L. Harlow

**Affiliations:** 1 Department of Biology, Texas A&M University, TAMU 3474, College Station, 77843‐3474, Texas; 2 Assistant Professor of Biology, Texas A&M University, TAMU 3474, College Station, 77843‐3474, Texas

**Keywords:** Neuromuscular junction, Single‐molecule detection, synaptic transmission, synaptic vesicle

## Abstract

The type of neurotransmitter secreted by a neuron is a product of the vesicular transporters present on its synaptic vesicle membranes and the available transmitters in the local cytosolic environment where the synaptic vesicles reside. Synaptic vesicles isolated from electroplaques of the marine ray, *Torpedo californica*, have served as model vesicles for cholinergic neurotransmission. Many lines of evidence support the idea that in addition to acetylcholine, additional neurotransmitters and/or neuromodulators are also released from cholinergic synapses. We identified the types of vesicular neurotransmitter transporters present at the electroplaque using immunoblot and immunofluoresence techniques with antibodies against the vesicle acetylcholine transporter (VAChT), the vesicular glutamate transporters (VGLUT1, 2, and 3), and the vesicular nucleotide transporter (VNUT). We found that VAChT, VNUT, VGLUT 1 and 2, but not 3 were present by immunoblot, and confirmed that the antibodies were specific to proteins of the axons and terminals of the electroplaque. We used a single‐vesicle imaging technique to determine whether these neurotransmitter transporters were present on the same or different populations of synaptic vesicles. We found that greater than 85% of vesicles that labeled for VAChT colabeled with VGLUT1 or VGLUT2, and approximately 70% colabeled with VNUT. Based upon confidence intervals, at least 52% of cholinergic vesicles isolated are likely to contain all four transporters. The presence of multiple types of neurotransmitter transporters – and potentially neurotransmitters – in individual synaptic vesicles raises fundamental questions about the role of cotransmitter release and neurotransmitter synergy at cholinergic synapses.

## Abstract (precis)

Synaptic vesicles isolated from electroplaques of the marine ray, *Torpedo californica*, have served as model vesicles for cholinergic neurotransmission. We found that greater than 85% of the cholinergic vesicles colabeled for a glutamatergic transporter, and approximately 70% colabeled with a nucleotide transporter; at least 52% of cholinergic vesicles isolated are likely to contain all four transporters.

## Introduction

Neurons are often classified by the small molecule neurotransmitters they release from synaptic vesicles along their axons and axon terminals. The type of neurotransmitter loaded into a synaptic vesicle is a product of the neurotransmitter transporters present in a vesicles membrane, combined with the local concentration of neurotransmitter and availability of cofactors necessary for active transport. Thus, at a cholinergic synapse, the presynaptic bouton will contain the precursors and enzyme machinery to produce acetylcholine (ACh) (Tuček 1988), and the synaptic vesicles present will contain the vesicular acetylcholine transporter (VAChT) (Erickson et al. 1994).

The simple idea that each neuron secretes only one type of neurotransmitter has been thrown into doubt with examples of cholinergic, GABAergic (gamma‐Aminobutyric acid) and noradrenergic neurons that appear to corelease glutamate (El Mestikawy et al. 2011; Hnasko and Edwards 2012). However, whether this corelease involves packaging of multiple transmitters into single synaptic vesicles is less clear. This is an important issue because it impacts how one thinks about the communication occurring at the synapse. Synaptic vesicles isolated from electroplaques of the marine ray, *Torpedo californica*, have previously been shown to contain the VAChT, as well as load and release the neurotransmitters (ACh) and adenosine triphosphate (ATP) (Dowdall et al. 1974). Thus, in the case of the electroplaque, ACh and ATP are either in the same synaptic vesicles, or two populations of vesicles reside in the terminals. Additionally, it has been demonstrated that synaptosomes from electroplaque release glutamate (Vyas and Bradford 1987), in agreement with studies that show some cholinergic neurons within the central nervous system (CNS) synthesize one or more vesicular glutamate transporters (VGLUT 1,2,3) and release glutamate at their terminals (Herzog et al. 2004; Gras et al. 2008; Ren et al. 2011). Two nonmutually exclusive possibilities could account for cases where corelease has been demonstrated. Either these neurons have a heterogeneous population of two or more distinct types of vesicles containing different neurotransmitter transporters, or these neurons possess a homogenous population of synaptic vesicles that contain multiple types of neurotransmitter transporters.

To address the question of whether at one type of synapse the synaptic vesicles are heterogeneous or homogenous, we have chosen to use vesicles isolated from the electroplaques of *T. californica*. The electroplaque is a classic preparation that provides an abundance of cholinergic synaptic vesicles from one class of motor neuron (Whittaker 1984). Two types of neurotransmitter are stored in and released from vesicles at these terminals – acetylcholine and ATP. In addition to the neurotransmitter transporters for acetylcholine and ATP (Sawada et al. 2008), we tested for the presence of glutamate transporters VGLUT 1, 2, and 3, (Bellocchio et al. 2000; Takamori et al. 2000, 2002; Varoqui et al. 2002; Shigeri et al. 2004); which are coexpressed in other cholinergic neurons. Western blot analysis confirms the presence of four neurotransmitter transporters in the isolated vesicle preparation, immunofluorescence labeling on cryostat tissue sections of electroplaques is consistent with the immunogenic epitopes of the four neurotransmitter transporters residing in the “cholinergic” axons and presynaptic terminals, and total internal reflection fluorescence (TIRF) (Axelrod et al. 1984) experiments on single vesicles (Mutch et al. 2011a) provide evidence that individual synaptic vesicles likely contain four types of neurotransmitter transporters. Therefore, we conclude that vesicles in the axon and axon terminals of the electroplaque are a homogenous population containing at least four types of neurotransmitter transporters and three different neurotransmitter/neuromodulators including acetylcholine, ATP, and glutamate.

## Material and Methods

### Reagents

Mouse monoclonal antibodies to VGLUT1 (N28/9), VGLUT2 (N29/29), VGLUT3 (N34/34), as well as the mouse monoclonal antibody for VAChT (N6/38) were purchased from Antibodies Incorporated (UC Davis/NIH NeuroMab Facility, Davis, CA). Additional antibodies included vesicular nucleotide transporter (VNUT) rabbit polyclonal (ABN110), synaptophysin rabbit polyclonal (MAB5258), Goat Anti‐Rabbit horseradish peroxidase (HRP), and Goat anti‐muse HRP (Millipore, Billerica, MS), and VAChT rabbit polyclonal ab68986 (Abcam, Cambridge, England) Fluorescently tagged secondary antibodies included Pacific Blue Goat anti‐mouse (P31582), Alexa 488 Goat anti‐rabbit (A‐11034), Alexa 488 Goat anti‐Mouse (A‐10667) (Invitrogen/Life Technologies, Carlsbad, CA). Alexa 594 *α*‐bungarotoxin (B‐13423), and FM 4‐64 (T‐13320) were used to label AChR and vesicles, respectively (Invitrogen). Pro‐long gold anti‐fade (P36934) was used to mount specimens (Invitrogen). Blue/green calibration beads (100 nm) were from Zeiss (Carl Zeiss, Oberkochen, Germany). All other reagents were purchased from Sigma‐Aldrich (St. Louis, MO) unless otherwise noted.

All figures were adjusted using ImageJ (NIH, Bethesda, MS). Schematic drawings and figure layout and labeling were done with Adobe Illustrator and Photoshop. (Adobe Systems Inc., San Jose, CA.)

### Isolation and enrichment of cholinergic vesicles

Methods were adapted from Ohsawa et al. (1979). A Spex Freezer Mill 6800 (Spex Sample Prep, Metuchen, NJ) was cooled to −180°C and ~25 g of frozen electric organ from *T. californica* (Aquatic Research Consultants, San Pedro, CA), containing abundant stacks of electroplaque was ground with 25 g of frozen buffer pellets (320 mmol/L Sucrose, 10 mmol/L TRIS‐Cl, pH 7.4). The resulting powder of buffer and electric organ was placed in a beaker and warmed to 4°C with 50 mL of buffer solution (320 mmol/L Sucrose, 10 mmol/L Tris‐Cl, pH 7.4, 4°C). The resulting slurry (100 mL) was centrifuged at 20,000 rpm for 10 min (Beckman Coulter JA‐20 rotor – Avanti J25 centrifuge) (Beckman Coulter, Brea, CA). The resulting supernatant was centrifuged at 34,000 rpm for 40 min (Beckman Coulter 70ti rotor – Optima ×80 centrifuge). The supernatant from this last centrifugation was loaded onto an eight milliliters 0.6 mol/L or 1.2 mol/L sucrose step gradient (10 mmol/L Tris‐Cl, pH 7.4), then centrifuged at 48,000 rpm for 2 h (Beckman Coulter 70ti rotor – Optima ×80 centrifuge). The 4–5 mL fluffy layer, enriched in vesicles, was collected. A 2 mL sample of enriched vesicles was filtered using a 0.22‐*μ*m spin column (Spin‐x, Corning; Corning, NY) to remove any large debris or clusters of vesicles. The resulting filtrate was injected into a Pharmacia LC500 plus Fast Protein Liquid Chromatography (FPLC), and run through a 25‐cm 4% agarose bead column (Bioscience Beads, West Warwick, RI). The FPLC was eluted with a buffer solution (0.2 mol/L NaCl, 10 mmol/L HEPES (4‐(2‐hydroxyethyl)‐1‐piperazineethanesulfonic acid), pH 7.4) at a flow rate of 1.0 mL/min. The second major peak was collected, and the vesicles concentrated to a protein concentration of 1 mg/mL or 8 mg/mL (measured by Bradford Assay – Bio‐Rad) (Bio‐Rad Laboratories, Inc., Hercules, CA) using a Stirred Cell apparatus with a 100 kDa filter (PLHK02510, Millipore).

### Electron microscopy

A 5 *μ*L sample of enriched synaptic vesicles (20 mg/mL as determined by Bradford assay) was pipetted onto a slot grid that had been treated by glow discharge. Excess sample was removed by filter paper, and the grid was briefly washed in deionized water, followed by 2 sec staining with uranyl acetate (Ted Pella, Redding, CA), followed by another wash (Jahn and Maycox 1988). The slot grid was viewed with a JEOL 1200 JEOL Ltd., Akishima‐Shi, TKY Japan) operated at 100 kV. Images collected at 30,000× and 50,000× magnifications on a bottom‐mounted 3072 × 3072, slow scan, lens‐coupled CCD camera SIA 15C (SIA, Dulith, GA).

### Immunoblotting

Four micrograms of vesicle protein was diluted 1:1 with Laemmli buffer (Bio Rad). The samples were loaded onto a 4–20% Mini‐Protean TGXTM precast gel (Bio Rad) and resolved for 35 min at 200 V. Proteins were transferred to a polyvinylidene difluoride (PVDF) membrane (Bio Rad) and probed with antibodies to synaptophysin, VAChT, VNUT, VGLUT 1,2, or 3. Antibody binding was detected with horseradish peroxidase‐conjugated secondary antibodies and ECL Prime (GE Healthcare, Fairfield, CT). Chemiluminescence was quantified using a Chemicdoc XRS+ imager (Bio Rad).

### Fluorescent immunohistochemical labeling of frozen sections of electroplaque and Wide‐field fluorescence

Sections (30 microns thick) of electroplaque were cut with a cryostat (Leica, Solms, Germany), collected on slides, and stored at −80°C until processed for labeling. For labeling, slides were removed from the freezer and dried within a vacuum desiccator (Savant – Thermo Fisher Scientific, Waltham, MA) for 5 min. The slices were then fixed in 4% paraformaldehyde for 5 min, rinsed with phosphate buffer solution (PBS), and washed in 0.1 mol/L glycine in PBS for 1 h. Slides were extracted in 0.5% Triton PBS for 15 min on ice, and rinsed 3× for 10 min in PBS. The samples were then blocked in 3% bovine serum albumin (BSA) in PBS for 1 h. Primary antibodies were diluted 1:250 in 3% BSA PBS and incubated overnight at 4°C. After washing 3 × 15 min in 3% BSA 0.1% Tween‐20 PBS, the secondary antibodies (Alexa 488 Goat anti‐Mouse or Alexa 488 Goat anti‐rabbit) were diluted 1:1000 in 3% BSA and a 1:1000 Alexa 594 *α*‐bungarotoxin added and incubated for 2 h. The slides were washed 3 × 15 min in 3% BSA PBS, blotted, and mounted on a coverslip in Pro‐long Gold for imaging.

Images of the electroplaque were acquired using a Leica (Nussloch, Germany) epifluorescence microscope and a 40× (NA, 1.0) oil immersion objective, a CoolSnap HQ integrating CCD camera (PhotoMetrics, Huntington Beach, CA), and IPLab3.5 software (BioVision, Exton, PA) using a Macintosh computer (Apple Computer, Cupertino, CA). Filter sets used were standard HQ sets (#41001 for Alexa 488, and #41002 for Alexa 594; Chroma Technology, Rockingham, VT).

### Fluorescent immunohistochemical labeling of isolated synaptic vesicles and TIRF imaging of single vesicles

After a final pass through a 0.22‐*μ*m spin column (Spin‐x, Corning, Corning, NY) to remove any vesicles that may have become clustered during the stirred cell concentration step, 100 *μ*L of synaptic vesicles (1 mg/mL) were transferred into 300 *μ*L PBS (pH 7.4), and labeled with two rounds of primary and secondary antibodies following the procedure (Mutch et al. 2011a). Briefly, the vesicles were incubated for 4 h with 1 *μ*g of primary mouse antibody, incubated for 30 min with 20 *μ*L anti‐mouse IgG beads, briefly centrifuged, and the vesicle containing supernatant was then incubated with 0.5 *μ*g goat anti‐mouse secondary antibody labeled with Pacific Blue for 4 h. The vesicles were then incubated with 1 *μ*g primary rabbit antibody for 4 h, incubated for 4 h with 0.5 *μ*g goat anti‐rabbit secondary labeled with Alexa 488, and finally incubated with 20 *μ*L anti‐goat IgG beads. The beads were pelleted and the supernatant collected for imaging. The pairings of primaries were the following: VAChT mouse monoclonal and VNUT polyclonal, VGLUT1 mouse monoclonal and VAChT rabbit polyclonal, and VGLUT2 mouse monoclonal and VAChT rabbit polyclonal.

FM4‐64 (Gaffield and Betz 2006) was added to the labeled vesicles (final concentration 1 *μ*mol/L), and the samples were incubated on a glass bottom culture dish (MatTek P35G‐1.5‐20‐C, Ashland, MA) for 1 h prior to imaging to allow vesicles to settle on the coverslip. Culture dishes with settled vesicles were placed on a Zeiss Axio Observer Z1 Microscope with TIRF slider, 100× TIRF objective (NA 1.45). Images were acquired using AxioVision (Carl Zeiss, Oberkochen, Germany). Three separate images for each field were taken using laser lines and filter cubes paired to eliminate fluorescent cross talk between the dyes: laser line 401 with filter cube 73 high efficiency (HE) was used for Pacific Blue, laser line 488 with filter cube 38 HE was used for Alexa 488, and laser line 561 with filter cube 74 HE was used for FM 4‐64. Images were collected with a Roper S/W PVCAM EMCCD camera and analyzed using ImageJ (NIH) software. Suitable spots detected in the FM4‐64 channel were marked. The other channels were then quantified for label.

Confidence intervals for the binomial proportion were calculated using two‐sided Agresti and Coull (1998) confidence limits providing a statistical means to combine paired labeling experiments that otherwise would have been impossible due to secondary antibody specificity and optical isolation of the three color channels used in the TIRF microscopy.

## Results

### Synaptic vesicle enrichment

**Figure 1.**
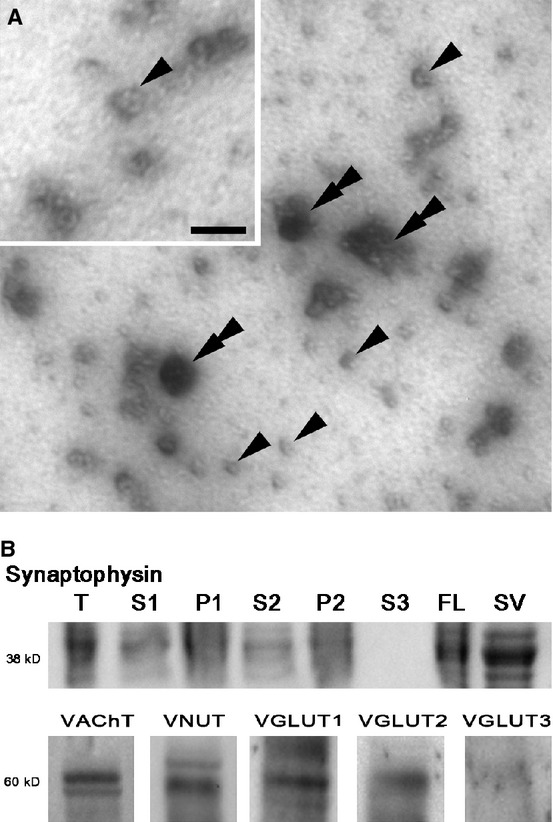
Synaptic vesicles can be enriched from electroplaques of *Torpedo californica* and shown to contain four neurotransmitter transporters. (A) Electron micrograph image (30,000×) of negatively stained vesicles isolated and enriched from electroplaque tissue. Sample contains abundant ~80 nm vesicles (some marked with single arrowheads) and occasional large clusters of vesicles and debris (double arrowheads). (A) (*insert*) Higher magnification (50,000×) negative stain image shows characteristic profile of membrane vesicles, Scale bar = 100 nm. (B) Immunoblot of the 38 kDa protein synaptophysin demonstrates isolation and enrichment of synaptic vesicles during the isolation procedure. Synaptophysin was seen in the tissue (T), and discarded pellets (P1, and P2), however, during isolation the majority of vesicles are maintained in the much larger by volume supernatant (S1 and S2) until the final centrifugation. In the final centrifugation, the vesicles move through the supernatant (S3) and collect on the fluffy layer (FL). The fluffy layer was collected, and the synaptic vesicles were further enriched by size exclusion chromatography (SV). Purified vesicles were tested by immunoblot and found to be positive for the ~60 kDa transporter proteins: VAChT, VNUT, VGLUT1, VGLUT2. Extra bands are possibly due to differences in protein glycosylation. No signal was detected for the transporter protein VGLUT3.

Synaptic vesicles were isolated from the electric organ of *T. californica* using standard vesicle isolation techniques. In order to verify that the synaptic vesicles were uniform and structurally intact, a sample of the vesicles was concentrated to 20 mg/mL, negatively stained (Jahn and Maycox 1988) and viewed with an electron microscope (Fig. 1A). In agreement with previous studies, the vesicles of *T. californica* are larger (80–120 nm) (Tashiro and Stadler 1978) than vesicles found at neuromuscular synapses in *T californica* (Fallon et al. 1985), or at neuromuscular synapses at other vertebrates (45–60 nm) (Nagwaney et al. 2009). Although single vesicle profiles were the dominant structure seen, some larger clusters were also present, and perhaps a result of the concentration procedure (Fig. 1A).

### Immunoblotting of neurotransmitter transporters

The electric organ of *T. californica* is derived from muscle fibers during development, and like all electrocytes, is a noncontractile muscle organ innervated by cholinergic neurons from the electric lobe. Fractions collected during the isolation procedure were immunoblotted for the synaptic vesicle protein synaptophysin to verify the isolation and enrichment procedure. Each lane contained the same amount of protein by Bradford Assay, however, pellet and supernatant volumes were not equal. As the isolation procedure advanced, synaptophysin was maintained and ultimately enriched in the final steps (Fig. 1B), providing further confirmation that the vesicular profiles seen by negative stain are from isolated synaptic vesicles (Wiedenmann and Franke 1985).

Immunoblotting was used to determine whether each of five types of neurotransmitter transporters representative of three types of neurotransmitters were present in the synaptic vesicles. As expected, synaptic vesicles isolated from the electric organ posses the cholinergic transporter VAChT (Fig. 1B). In addition to acetylcholine, it has previously been demonstrated that ATP is loaded and released by synaptic vesicles isolated from the electric organ (Whittaker et al. 1972). We tested for the presence of the purinergic ATP transporter in the vesicle preparation, and found it labeled for VNUT (Fig. 1B). In addition to testing for the transporters of known neurotransmitters at the electric organ, we probed for the three known glutamatergic transporters. Previous studies have demonstrated glutamatergic transporter expression in cholinergic neurons (Herzog et al. 2004; Gras et al. 2008), and in some cases glutamatergic signaling (Ren et al. 2011). By immunoblot, VGLUT 1 and 2, but not 3 was shown to be present in the synaptic vesicle preparation (Fig. 1B; supplemental).

**Figure 2.**
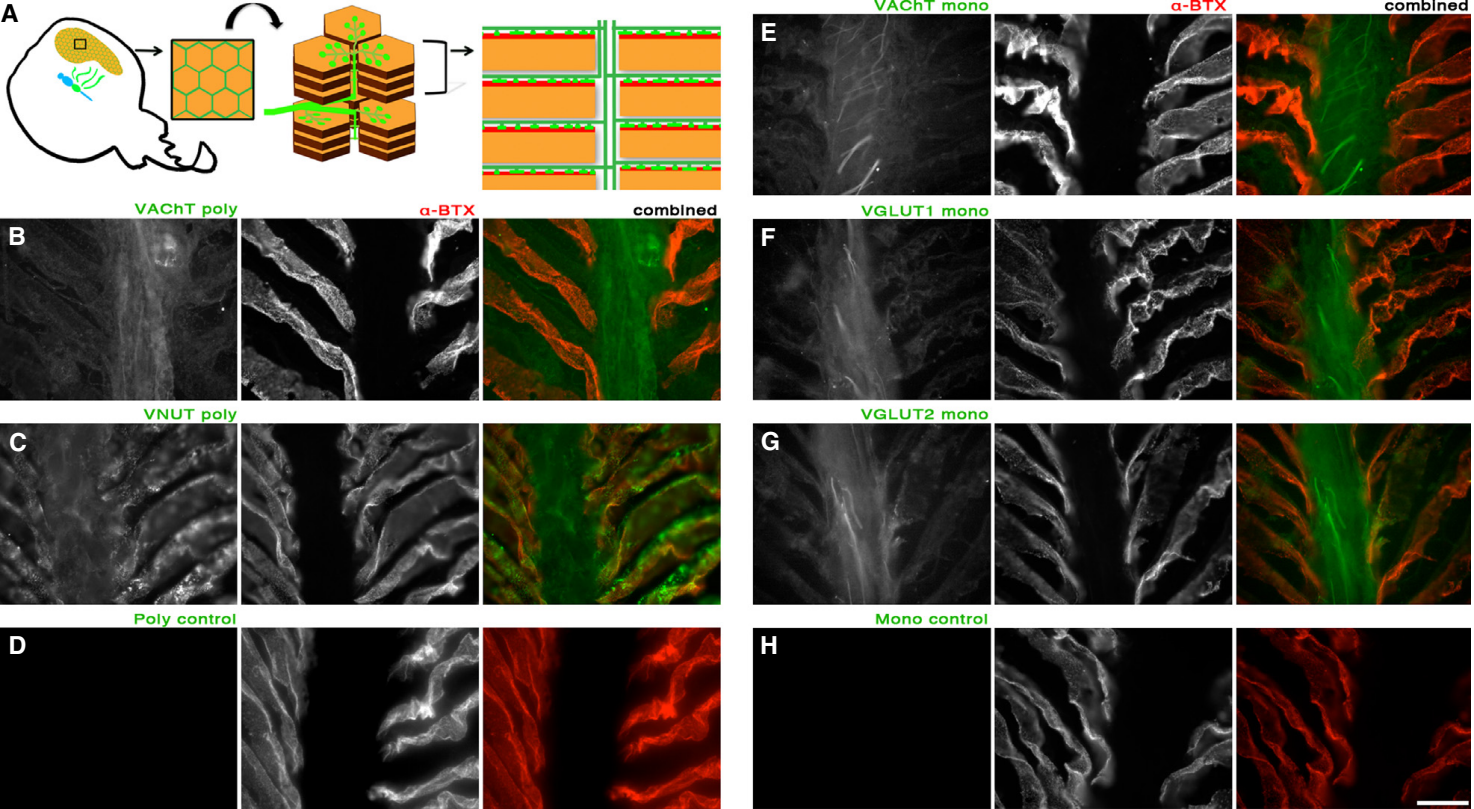
Antibodies against VAChT, VNUT, VGLUT1 and VGLUT2 stain axons and presynaptic boutons residing within the electric organ. (A) Cartoon depicting the electric organ of *Torpedo californica* (1 side of the paired organ shown) and its innervation by four electromotor nerves that project from the electric lobe of the central nervous system (green). From the surface, the electroplaque cells of the electric lobe appear as a honeycomb. Viewed from the side the electroplaque cells appear as large, pancake‐like stacks. Each individual electroplaque has a top surface covered in nicotinic acetylcholine receptors (red surface). That surface is richly innervated with presynaptic boutons and axons. Axons run in between the pancake stacks before entering the target electroplaque and forming synapses. The drawing to the far right in A illustrates the view shown in the remainder of the figure; however, in the immunostains, the receptor‐rich surface of each electroplaque commonly runs at an angle to the central core containing only axons. (B–H) Labeling with each of the neurotransmitter transporter antibodies was done in conjunction with *α*‐Bungarotoxin to mark the postsynaptic surface on the electroplaque cells. (B) VAChT polyclonal antibody, and (C) VNUT polyclonal antibody. (D) Application of a secondary antibody to tissue to which no primary polyclonal was applied. (E) VAChT monoclonal antibody, (F) VGLUT1 monoclonal antibody (G) VGLUT2 monoclonal antibody, and (H) secondary antibody in a control to which no primary monoclonal antibody was applied. Axons and presynaptic labeling can be seen in (B, C, and E–G) but no appreciable label was detected in the controls (D and H). Scale bar = 5 microns.

As a further verification of antibody specificity, we labeled cryostat sections of the electric organ. An electric organ, present on either side of the ray, is made up of pancake‐like stacks of noncontractile, muscle‐derived, cells termed electroplaques (Fig. 2A). On the top surface of each electroplaque is a high concentration of nicotinic acetylcholine receptors. Four large nerve bundles originate from the electric lobe, and axons from these nerves travel between the stacks of electroplaque cells before turning 90 degrees and innervating the entire surface of the electroplaque cell with presynaptic boutons. We found that VNUT, VGLUT 1 and 2, and VAChT labeled the axons (Fig. 2B, C, and E–G). In addition, we found labeling for each of the transporters was present above the postsynaptic surface of the electroplaque (labeled with *α*‐Bungarotoxin) (Fig. 2B, C, and E–H).

### Single‐vesicle imaging with TIRF microscopy

**Figure 3.**
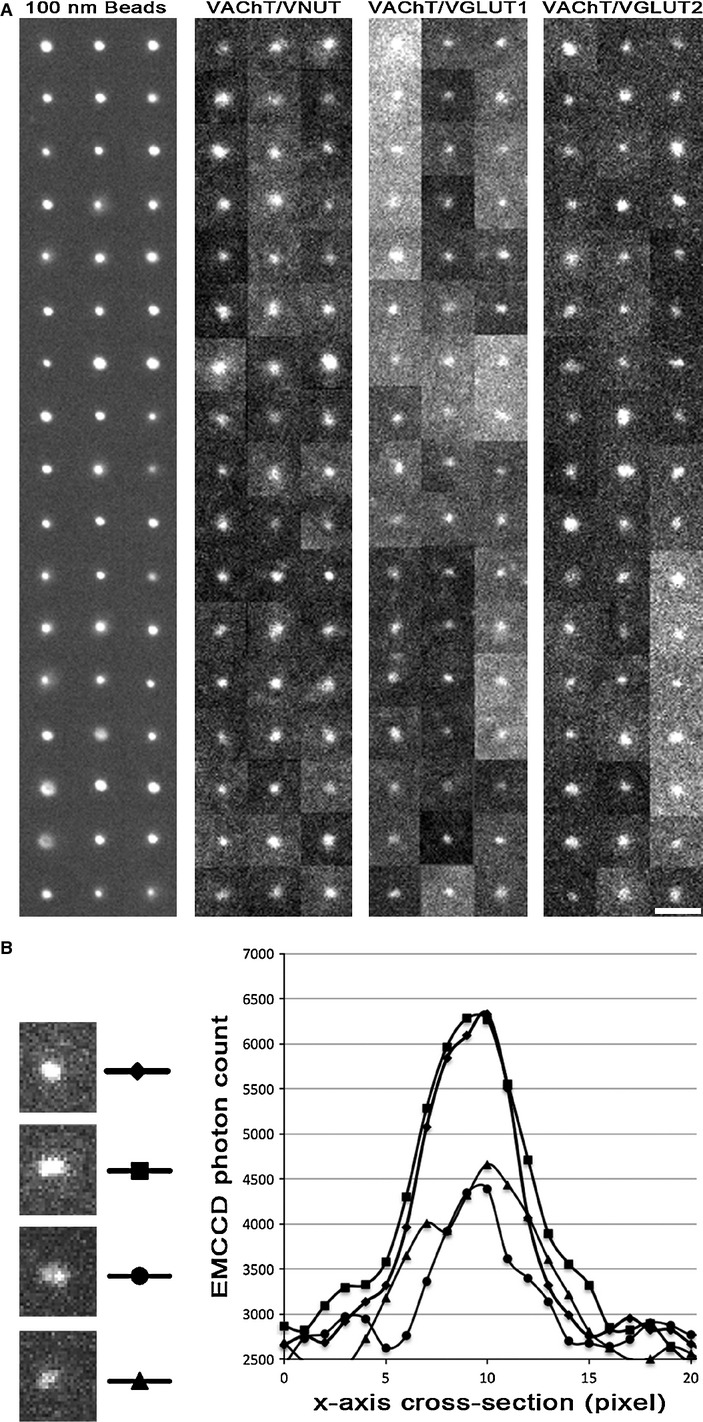
TIRF microscope images of vesicles labeled with FM4‐64 were used to verify the size and intensity of the spots matched that of a single vesicle (A) Fluorescent images were collected from 51 fluorescent beads (100 nm diameter) and compared to 51 synaptic vesicles labeled with FM4‐64 from each of the fluorescent pairings in this study. Although the fluorescent beads are brighter, the beads and the vesicles possess similar sized diameters and point spread function widths. Scale bar = 1 micron. (B) The fluorescence intensity of each putative vesicle was quantified in order to verify that each subdiffraction limited spot did not contain multiple vesicles. If two vesicles reside adjacent to one another they may still appear as single spot with a similar cross‐sectional width in the *X*‐axis; however, the photon count in the FM4‐64 channel would equal the sum of the two vesicles. Cross sections along the *X*‐axis were taken of four vesicles (marked at the site of the line labeled by a diamond, square, circle, or triangle), The diamond and square spots contain twice the intensity of labeling as the circle and triangle spots in the image, and are thus likely to be two adjacent vesicles within the same subdiffraction‐labeled spot

**Figure 4.**
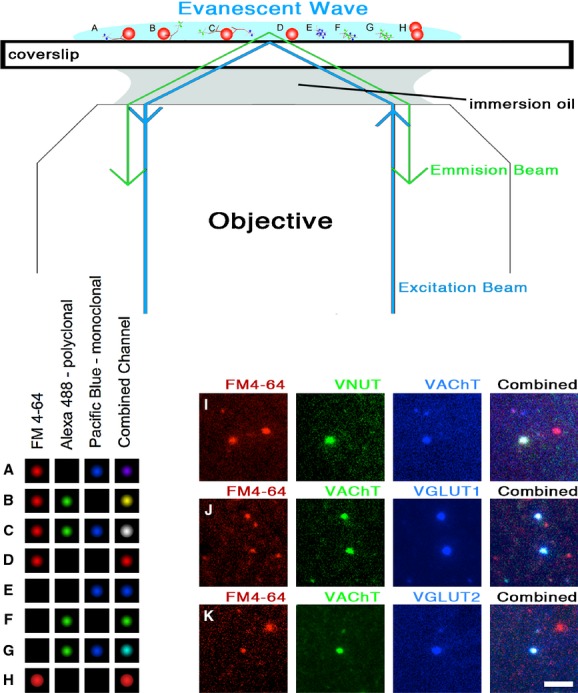
TIRF microscope images of single synaptic vesicles. During TIRF illumination, the excitation beam travels off‐axis through the microscope objective causing the beam's incident angle at the coverslip to be too shallow to allow the beam to pass into the sample, thus leading to the total internal reflection of the beam back into the objective. An evanescent field of illumination, approximately 100 nm in depth, is created at the surface of the coverslip, allowing for selective illumination of single molecules residing on the coverslip. A diagram of expected observations could include singly label vesicles A, B, or dually labeled vesicles **C**. FM4‐64 was used in order to control for three possible scenarios: (D) Nonlabeled vesicles or debris, (E–G) fluorescent secondary on the coverslip not associated with any synaptic vesicles, or (H) spots that contained brighter than standard labeling in the FM4‐64 channel, as discussed in Figure 3B, and were thus unlikely to be single vesicle events. Example TIRF images of dual labeled, single vesicle events for (I) VAChT and VNUT, (J) VAChT and VGLUT1, and (K) VAChT and VGLUT2. Scale bar = 1 micron.

Very little is known about the molecular components residing within individual synaptic vesicles, and virtually no information exists about the molecular homogeneity of vesicles within a nerve terminal. Recently a single‐molecule technique based upon total internal reflection fluorescence (TIRF) microscopy (Axelrod et al. 1984) has been developed that allows for the quantification of protein copy numbers present on single vesicles (Mutch et al. 2011b). In the CNS, the smaller glutamatergic vesicles (35 nm) were shown to possess four VGLUT1 transporters per vesicle (Mutch et al. 2011b). We chose to use this technique on vesicles isolated from the electric organ to address the question of whether single synaptic vesicles could have multiple classes of neurotransmitter transporters. Because the terminal is cholinergic, we chose to test the three other transporters VNUT, VGLUT1, and VGLUT2 in a pairwise labeling with the VAChT. The vesicle isolation procedure was designed to minimize vesicle clusters and larger cellular debris (Fig. 1A). In addition, the plating density was tittered to reduce the probability of two vesicles residing within a subdiffraction limited spot. In order to verify that single vesicles were imaged, the lipophilic dye FM4‐64 (Gaffield and Betz 2006) was added for two additional checks. For the first check, the resulting fluorescent spots (single vesicles) were checked to ensure they possessed a similar fluorescent profile as 100‐nm fluorescent control beads (Fig. 3A). For the second check, the fluorescence intensity of each spot was quantified in order to verify that each subdiffraction limited spot did not contain multiple vesicles (Fig. 3B). Although it was a rare event due to the titer density, we did occasionally find spots that had double or even triple the intensity expected for a single vesicle. Fluorescent profiles from the 4–64 channel that did not match the shape of a single molecule event (simple point spread function), or that possessed multiple quanta of lipid stain (as measured by fluorescence) were excluded from this study. Sites of FM4‐64 label that met the above criteria were then examined for the presence of secondary label in the two additional channels. One channel was optimized for the secondary label (Goat anti‐mouse) Pacific Blue, and the other secondary channel was optimized for the secondary label (Goat anti‐rabbit) Alexa 488. The types of possible outcomes are diagramed in Figure 4A–H. Instances of FM4‐64 label with no additional label in either of the other channels were not quantified. Instances of single channel label (Pacific Blue or Alexa 488), or instances of dual channel label (Pacific Blue and Alexa 488) were quantified, examples of which can be seen in Figure 4I–K.

**Figure 5.**
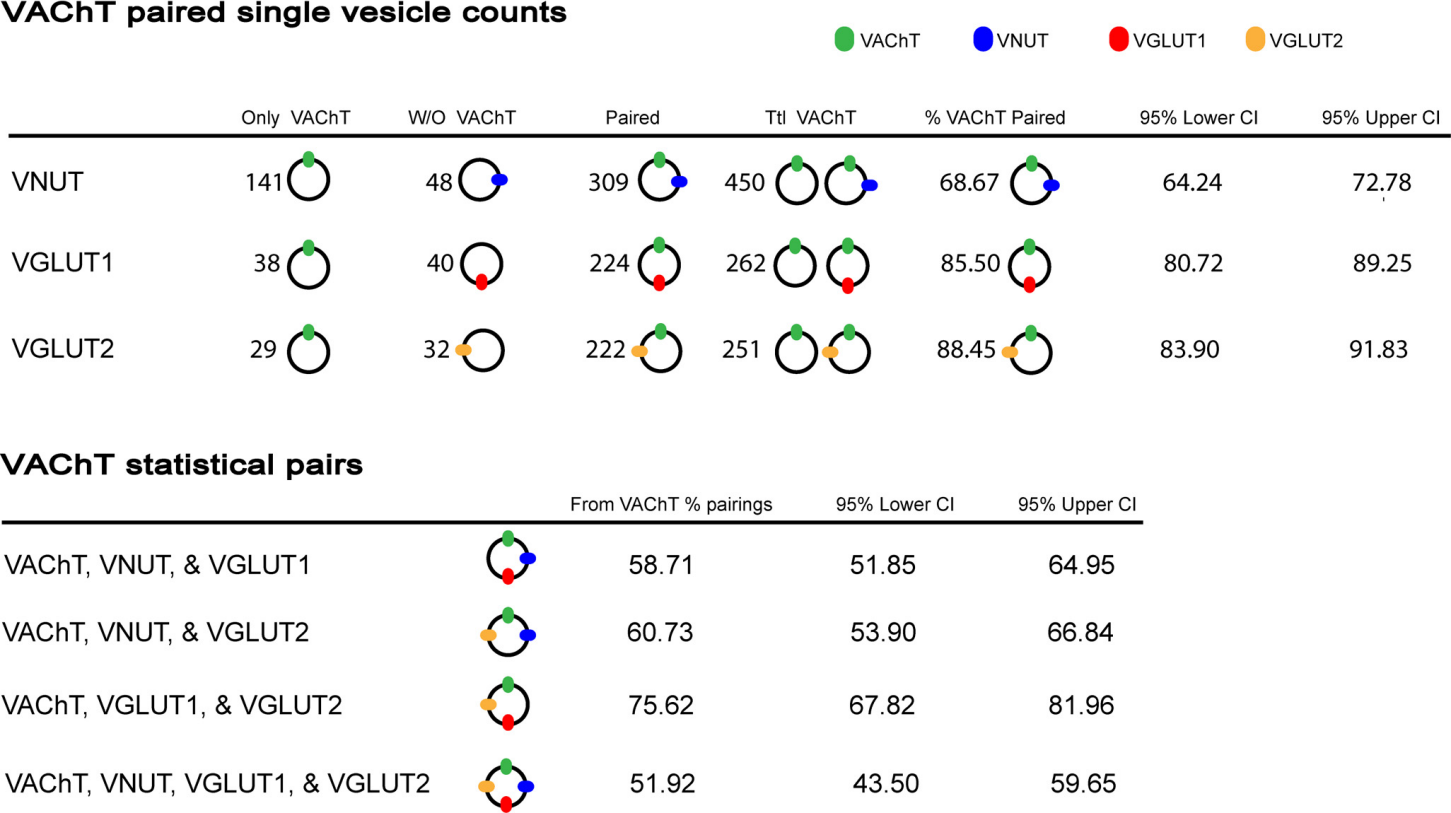
Single‐synaptic vesicle observations of vesicular transporters (VNUT, VGLUT1, or VGLUT2) colabeled with VAChT, and confidence intervals of statistically paired transporters calculated using two‐sided Agresti‐Coull confidence limits.

Statistical analysis of the VAChT‐labeled vesicles found a high degree of pairing in the second label channel, with a majority of the vesicles that labeled with VAChT also labeled with VNUT (69%), VGLUT1 (86%), or VGLUT2 (88%). Further statistical groupings can be quantified based on pairwise comparisons using Agresti Coull confidence limits (Agresti and Coull 1998). In addition to containing a VAChT, 59% of vesicles are likely to contain VNUT and VGLUT1, 61% of VAChT vesicles are likely to contain VNUT and VGLUT2, 75% of VAChT vesicles are likely to contain VGLUT1 and VGLUT2, and 52% of the single‐VAChT vesicles are expected to contain VNUT, VGLUT1, and VGLUT2 (Fig. 5).

## Discussion

In this study, we used single‐vesicle imaging to examine whether cholinergic synaptic vesicles possess additional types of neurotransmitter transporters. As observed by electron tomography, cholinergic vesicles at the frog's neuromuscular junction posses a stereotypic structure and arrangement of transmembrane molecules and appear homogenous (Harlow et al. 2013). We found that cholinergic vesicles isolated from the electric organ of *T. californica* have at least four different neurotransmitter transporters. In addition to the cholinergic transporter VAChT, a purinergic transporter VNUT and two glutamatergic transporters VGLUT1 and VGLUT2 were shown to colocalize with VAChT to individual vesicles.

Until recently, the stimulated release of acetylcholine and ATP from the motor neurons of the electric lobe (Dowdall et al. 1974) stood as one of the few exceptions to Eccles et al. (1954) interpretation of Dale (1935) hypothesis – one neuron, one neurotransmitter. Not only are the two neurotransmitters released from the presynaptic terminals in a stimulation‐dependent manner, ensemble measurements of synaptic vesicles isolated from *T. californica* clearly demonstrate that both neurotransmitters actively load into the synaptic vesicles. Subsequent work has demonstrated that ATP acts on specific purinergic receptors at terminals (Burnstock 2006), and the transporter responsible for loading ATP into the vesicles, VNUT, has been cloned and characterized (Sawada et al. 2008). However, prior to this study, whether individual synaptic vesicles contained both cholinergic and purinergic transporters was unknown. Using single‐vesicle imaging we demonstrate that at least 69% of the VAChT containing vesicles also posses VNUT, indicating that indeed individual vesicles do contain both cholinergic and purinergic transporters and are therefore most likely filled with and secrete both ACh and ATP simultaneously.

Glutamate is perhaps the most abundant neurotransmitter in the nervous system of vertebrates. Based upon our initial transcriptome of the electric lobe, we believe homologs for all three glutamate transporters are present in *T. californica* (data not shown). However, neuromuscular junctions in vertebrates like *T. californica* utilize acetylcholine as their primary excitatory neurotransmitter. Still, many of the conditions for considering glutamate as a neurotransmitter, or at least a neuromodulator, at cholinergic motor neurons have been met. Glutamate plasma membrane transporters used to remove glutamate after release are present in muscle cells, and in fact concentrated in the folds of the neuromuscular junction just opposite the presynaptic release site (Rinholm et al. 2007). Glutamate has been shown to activate a nitric oxide signaling pathway in muscle cells (Urazaev et al. 1995) that in turn modulates both the number of vesicles released presynaptically (Pinard et al. 2003) and the amount of acetylcholine released by nonquantal means (Malomouzh et al. 2003). Glutamate release from the presynaptic terminal has not been demonstrated at the vertebrate neuromuscular junction, although glutamate release has been demonstrated from lower motor neurons onto Renshaw cells (Herzog et al. 2004), and from synaptosomes isolated from *T. californica* (Vyas and Bradford 1987). We find that at least 86% of the VAChT‐containing vesicles also posses VGLUT1, and 89% posses VGLUT2. At this time, we cannot exclude the possibility that VGLUT3 might be present in these vesicles, and that the antibody used in this study was not specific to the homolog in *T. californica*. While the synapses at electroplaque cells are not contractile, the possibility that a vesicular glutamate transporter may also be present at vertebrate neuromuscular junctions warrants further study.

The results presented in this paper show single synaptic vesicles that possess two or more neurotransmitter transporters – with at least 52% of vesicles possessing all four neurotransmitter transporters (Fig. 5). Assuming more than one type of neurotransmitter to be present in the terminal, fusion of one of these vesicles with the plasma membrane would produce corelease of two or more neurotransmitters. In addition to the ability to load more than one neurotransmitter, the transporters, and the neurotransmitters themselves, may alter the proton gradient (ΔpH) and membrane potential ΔΨ across the vesicle membrane, and in fact may work in a synergistic manner (El Mestikawy et al. 2011; Hnasko and Edwards 2012). Ensemble measurements of acetylcholine loading into isolated CNS cholinergic vesicles show a marked increase in the presence of glutamate (Gras et al. 2008). Glutamate is known to increase the ΔpH across the vesicle membrane, and ΔpH is the most important driving force for VAChT activity. With a negative charge at cytosolic pH, ATP would also provide an increase in ΔpH, although an exact measurement of that activity would be difficult given the requirement of ATP for the vesicular proton pump. The presence of multiple types of neurotransmitter transporters, taken as a whole, provide a mechanism to explain cotransmitter release, and in the case of cholinergic vesicles, synergistic neurotransmitter loading effects.

## Acknowledgments

We are grateful for the microscopy expertise of Rola Mouneimne, and for the helpful criticisms of the manuscript from Kirsten Bohn, Joseph Szule and Matthew Lee.

## Conflict of Interest

None declared.
